# Co-Designed Digital Device for Tracking Rehabilitation Dosage in a Clinical Environment After Stroke: Mixed Methods Validity and Feasibility Study

**DOI:** 10.2196/68129

**Published:** 2025-05-14

**Authors:** Fiona Boyd, Gillian Sweeney, Mark Barber, Elaine Forrest, Mark Dunlop, Andrew Kerr

**Affiliations:** 1 Department of Biomedical Engineering, University of Strathclyde, Wolfson Centre, 106 Rottenrow, Glasgow, G4 0NW, United Kingdom, 44 (0) 141548310; 2 Occupational Therapy Department, University Hospital Wishaw, NHS Lanarkshire, Wishaw, United Kingdom; 3 Department of Medicine for the Elderly, Monklands Hospital, NHS Lanarkshire, Airdrie, United Kingdom; 4 Department of Computer and Information Sciences, University of Strathclyde, Glasgow, United Kingdom

**Keywords:** stroke, digital tracking, rehabilitation technology, design, user-centered, digital health, digital technology, digital interventions, assistive technology, wearable health, health care innovation, therapy adherence, therapeutic dosage tracking, rehabilitation tracking, usability

## Abstract

**Background:**

In 2023, the National Clinical Guidelines for Stroke revised the recommended daily multidisciplinary therapy dose from 45 minutes per therapy to 3 hours of therapy overall. To monitor the achievement of these guidelines, there is a need for accurate measurement. This study introduces a novel co-designed digital dosage tracking system that uses Near Field Communication technology to log rehabilitation activities and demonstrates its feasibility and accuracy in a clinical setting through comparison with the current clinical method of manual recording.

**Objective:**

This study aimed to assess the validity, feasibility, and usability of a novel co-designed digital tracker using Near Field Communication technology to automatically log rehabilitation dosage in people with stroke history, providing an objective and low-burden solution for clinical environments.

**Methods:**

This pilot mixed methods study included 2 phases. Phase 1 involved a usability trial with 9 participants conducted at a university research center, assessing usability with the System Usability Scale (SUS) and Intrinsic Motivation Inventory (IMI). Phase 2 consisted of a clinical trial in a National Health Service stroke ward with 15 inpatients, comparing the digital tracker with manual therapist recordings for validity and feasibility using paired *t* tests, Cohen *d*, and Bland-Altman plots. An acceptable discrepancy range was set at ±5%‐10%.

**Results:**

The digital tracker demonstrated high usability with a mean SUS score of 91.43 (SD 9.53) and strong user satisfaction (IMI score 6.29/7, SD 1.50). Clinical trial results showed a strong agreement between the digital and manual methods (*t*_206_=−1.60; *P*=.11; Cohen *d*=−0.06), with a small mean time discrepancy of 1.23 (SD 11.01) minutes across 207 activities. The Bland-Altman plot indicated good accuracy and consistency between methods, with limits of agreement within the clinically acceptable range.

**Conclusions:**

The co-designed digital tracker has been shown to agree with a manual method for recording rehabilitation dosage. This development presents the opportunity for objective, automated, and low-burden recording of rehabilitation dose to support prescription, monitoring, and research.

## Introduction

Rehabilitation is crucial for regaining functions lost to stroke. The National Clinical Guidelines for Stroke in the United Kingdom and Ireland now recommend at least 3 hours of daily multidisciplinary therapy, a fourfold increase from the previous 45 minutes [1]. Yet, in practice, people often receive a total of 35.6 minutes per day of in patent stay therapy [2,3]. These shortfalls were attributed to highly varied nurse and therapy staffing levels exacerbated by understaffing issues, and organizational factors such as the timing of therapy assessments and the presence of weekend therapy services. In addition, less than half of stroke teams provided an extended weekend therapy service.

Stroke rehabilitation activity in the United Kingdom is measured through manual tracking, typically based on therapists’ notes. The Sentinel Stroke National Audit Programme (SSNAP) database is a national stroke register that audits care from approximately 250 stroke teams in England, Wales, and Northern Ireland through a combination of electronic data entry by the stroke team and manual data inputting to the SSNAP’s online clinical database. Whilst SSNAP captures daily therapy duration and other key metrics, it lacks data on the structure, content, and timing of therapy sessions, and inconsistencies in therapist-reported durations have been noted with a tendency to overestimate the duration of treatment sessions [4]. This absence of standardized, automated, and objective methods for monitoring rehabilitation dosage and intensity hinders adherence to guidelines, personalized care, and research into innovative interventions.

Rehabilitation dosage refers to the total amount of therapy delivered, encompassing the intensity, frequency, and duration of rehabilitation activities [5]. Intensity, as defined by the United Kingdom’s National Institute for Health and Care Excellence (NICE) guidelines, involves tailoring the amount of therapy each day to meet the individual’s needs across various domains, such as physiotherapy, occupational therapy, and speech and language therapy [6]. In the National Health Service (NHS), the dosage of stroke rehabilitation a participant receives is typically recorded manually by health care professionals, who document the time spent undertaking their rehabilitation during a therapy session, the specific activities performed, and the intensity of these activities in relation to the amount of therapy each day to meet the individual’s needs [7]. This manual recording process is regulated for auditing and developing stroke care pathways [8].

The use of Internet of Things (IoT) and mHealth devices for self-rehabilitation shows promise in improving post-stroke recovery and quality of life [9]. Near-Field Communication (NFC) has been proposed as a means of recording patient information [10], yet there remains limited evidence of its use for clinically tracking acute or subacute stroke rehabilitation dosage or intensity. One study measured stroke dosage through electronic counters that required participants to press a button after each repetition [11]. While a promising approach, this places the burden of measurement on the participant. Home-based rehabilitation tracking using computers and tablets have also been investigated, particularly in post-stroke aphasia, and been suggested as a potential solution to standardizing dose measurement [12-14]. However, studies proposing electronic or digital methods for dosage tracking often do not adopt a co-design approach that would accommodate the full range of stroke severities, including cognitive and aphasic impairments. According to Medical Research Council recommendations [15], the usability of rehabilitation tracking technology should be driven by a collaborative process involving both users and health care professionals, ensuring that design and functionality are optimized to address diverse user needs.

This study introduces a novel, co-designed tracker that uses NFC technology to track the dosage of rehabilitation across multiple domains (physical, cognitive, and communication). The tracker was designed with users (stroke survivors and stroke rehab professionals) to be accessible, clinically appropriate, and easy to implement, providing a comprehensive and user-friendly solution for monitoring compliance with stroke rehabilitation guidelines.

## Methods

### Overview

This pilot study, conducted in 2 phases, evaluated the digital dose tracker, co-designed by users, by assessing its feasibility, validity, and usability. Phase 1 focused on a usability study (n=9) conducted in a research setting. Phase 2 involved a clinical trial to assess the trackers’ feasibility and validity (n=15). The primary outcome for Phase 1 was usability, measured by the System Usability Scale (SUS) and Intrinsic Motivation Inventory (IMI). The primary outcomes for Phase 2 were dose validity (comparison with the current method) and tracker feasibility [16]. Feasibility was defined as the ability of the digital dose tracker to be effectively used by all participants, including those with cognitive or physical impairments, without requiring significant modifications or support. It was assessed by the absence of user errors in Phase 2, withdrawals, and the successful capture of accurate rehabilitation data across various activities [17].

### Co-Design of Digital Dose Tracker

Two focus groups were conducted; first, to determine the need for a digital tracker and brainstorm ideas with the stroke rehabilitation community, second to create the product design criteria and user requirements for both hardware and software [16]. The same participants (6 stroke survivors and 1 physiotherapist) were involved in both focus groups. Design requirements were extracted through thematic analysis and ranked by priority which was determined by the frequency of mentions paired with the number of participants contributing. Once the design criteria were prioritized this was verified with the focus group participants. The key outcomes of these focus groups were the clear need to create a device that could track both dosage and intensity but was simple to use and could motivate engagement with rehabilitation [18].

### Device Specifications

**Figure 1.**
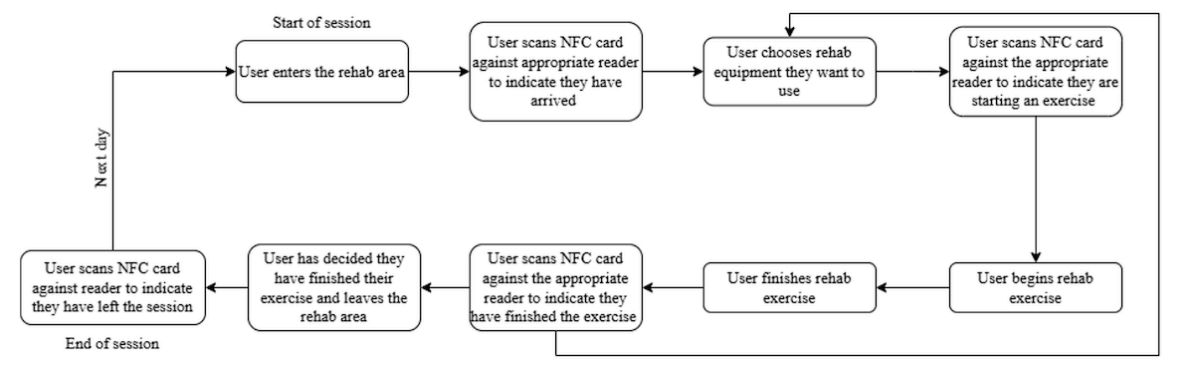
The user journey of the digital dose tracker. NFC: Near-Field Communication; rehab: rehabilitation.

A recommendation from the cocreation process was to use a contactless ID card reader positioned at various rehabilitation workstations (Figure 1) [19,20], enabling users to tap their card and self-track their dosage in real time.

**Figure 2.**
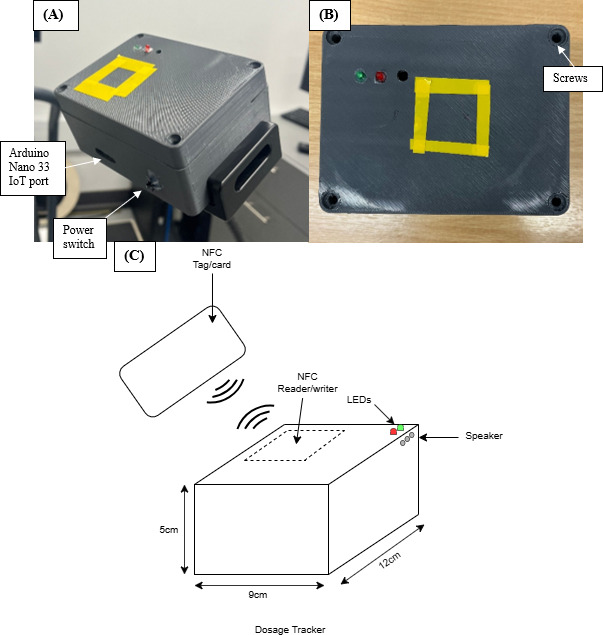
Photographs and diagram of the near-field communication dosage tracker. (A) Overall view of the tracker, (B) top-down view, and (C) diagram illustrating key components and functionality. IoT: Internet of Things; NFC: Near-Field Communication.

**Figure 3.**
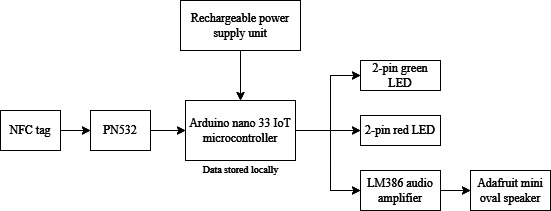
System block diagram. IoT: Internet of Things; NFC: Near-Field Communication.

The dosage trackers (Figure 2) consisted of an Arduino Nano 33 IoT (SAMD21, Arduino, Italy), a PN532 NFC module (NXP Semiconductors, Netherlands), green and red LEDs and an Adafruit mini speaker (Adafruit Industries; Figure 3) to provide visual and audio feedback. A rechargeable battery (5 volts, 1 amp) provided the power. Each tracker was programmed (C++) to register a participant’s unique identifying number when they scanned their NFC card, allowing each rehabilitation activity and the corresponding time spent at each workstation, to be attributed to the correct user. Figure 2 highlights the positions of the light-emitting diodes, speaker openings, and the Near-Field Communication reader and writer. In addition, it depicts a Near-Field Communication card being brought into proximity with the device to facilitate communication between the card and the reader for logging participant dosage. The yellow markings in Figure 2A and 2B serve as visual guides to indicate the precise location of the Near-Field Communication reader, minimizing scanning errors.

When a user places their unique NFC card near a tracker for the first time, the green LED flashed once, and the speaker emitted a tone indicating a successful “scan in” to the equipment or room. Holding the card near the tracker a second time caused the green LED to flash twice, accompanied by another tone, confirming a correct “scan out.” If a user then presented the same card to the tracker again, the system recognized that the same piece of equipment was being reused, or the same room was being re-entered. The card not being placed close enough to the tracker or being moved away too quickly could create registration errors. In these instances, the red LED flashed 3 times, and the speaker played an error sound, prompting the user to re-scan the card.

### Phase 1: Usability

#### Participants

The usability of the digital dosage tracking system was tested with 9 participants recruited from an ongoing trial (ClinicalTrials.gov; NCT06787768) at the Sir Jules Thorn Centre for Co-Creation of Rehabilitation Technology, University of Strathclyde [17,18]. That trial was investigating the feasibility of a technology-enriched rehabilitation gym for people living with stroke, delivered entirely through technology (eg, virtual reality, treadmills, weight suspension, and power assistance equipment) over an 8-week program. Individuals in the trial were required to have experienced a stroke at least 12 months before, be in stable health, and be able to attend at least 2 of the 2-hour sessions per week, follow instructions in English, and offer verbal or written feedback. Within the technology-enriched rehabilitation gym, a physiotherapist designed, supervised, and reviewed each participant’s program using principles of intensity, feedback, cognitive engagement, and aerobic activity, aligning it with the participant’s goals and baseline outcome measures. Since all 9 participants met these criteria, they were also eligible to join our 8-week usability study, providing a controlled environment to evaluate the digital dosage tracking system.

#### Testing

Participants attended rehabilitation sessions in the research facility, where they used the trackers to log their rehabilitation activities. The activities and workstations used during this usability trial were matched to those in the subsequent clinical trial, maintaining consistency across both study phases. After completing the 8-week usability trial, participants filled out the SUS and IMI surveys to assess their experience with the system.

#### Analysis

The data gathered from the SUS and IMI surveys were analyzed to determine the usability of the digital dosage tracking system. The SUS provided a quantitative measure of usability, whilst the IMI offered insights into the participants’ motivation and engagement with the system. The results were used to evaluate the trackers potential for integration into clinical practice

### Phase 2: Feasibility and Validity

#### Participants

In-patients at a local NHS hospital, with a diagnosis of stroke and referred for rehabilitation, were invited to participate in the study, provided they met the following inclusion criteria: diagnosis of a new stroke by an NHS Lanarkshire physician, more than 48 hours since the stroke event, medically fit for rehabilitation as determined by medical staff, deemed to require rehabilitation, and able to provide informed consent. Individuals were excluded if they were acutely medically unwell, had active cardiac disease (such as unstable angina), active delirium or significant levels of confusion, had a seizure within the past 7 days, were being managed under the Adults with Incapacity Act, or were known to be pregnant. Those with aphasia and other cognitive or communicative difficulties resulting from their strokes were not excluded from participating. Given the nature of the stroke population, a significant proportion of participants were likely to have these impairments. In the United Kingdom, it is estimated that 50% of stroke survivors experience cognitive impairment, and approximately one-third have aphasia or other communication difficulties, particularly in the acute and sub-acute phases of recovery [21].

#### Testing

Participants attended as many sessions at a rehabilitation gym located on the stroke ward as able, with sessions lasting up to 2 hours. The tracker recorded the start and stop times when a participant scans their card at each activity, providing a log of the total time spent at each activity, the person who used it and exactly what the activity was to a local database based within the ward. At the same time, 2 supervising clinical staff members observed all participants and manually recorded the rehabilitation activities and durations. This manual logging was intended to simulate typical clinical practice, based on the advice of the local rehabilitation team, where therapists record the time spent at each station or device without tracking finer details such as repetitions or specific exercises.

#### Analysis

To assess the equivalence of the digital dosage tracking system with manual recording methods, paired sample *t* tests were used to compare the mean times recorded by the 2 methods for each activity. Cohen *d* was calculated to quantify the effect sizes of any observed differences, with small effect sizes being indicative of negligible differences. Bland-Altman plots were used to visually explore the relationship between the 2 methods, and the limits of agreement (LOA) were calculated with a 95% CI to determine the range within which most differences would fall. Time recordings were standardized to minutes, and the average absolute time discrepancies and their variability are presented in the Results section. An acceptable range for discrepancies was decided to be within ±5%.

### Ethical Considerations

This study received ethical approval from the Strathclyde University Ethics Committee under the protocol number UEC20/08: Kerr: Generic Framework Application: User experience of a technology-based rehabilitation program. All participants provided informed consent before their involvement in the study. Participants did not receive compensation, and all data were anonymized. The study was part of a clinical trial (ClinicalTrials.gov; NCT05981729) and received NHS Research & Development ethical approval from South East Scotland Research Ethics Committee 1 (IRAS ID No. 329156).

## Results

### Phase 1: Usability

A total of 9 people participated in phase 1 of this study (Table 1); however, 2 participants decided to withdraw from the trial in the final week, 1 due to other commitments and the second due to short-term illness. High usability was demonstrated with a mean IMI score of 6.29/7 (SD 1.5; Table 2), and a mean SUS score of 91.43/100 (SD 9.53); the total SUS score was calculated based on participants’ responses to the 10-item questionnaire, with each item rated on a 5-point Likert scale. The scores were normalized and converted to a range of 0-100 (refer to Table 3 for further SUS results).

**Table 1.** Participant characteristics of usability trial.

Characteristics	All participants (n=9)
Age (years), mean (SD)	62.56 (10.32)
Sex, n	
Male	8
Female	1
Aphasia, n	
Aphasic	2
Non-aphasic	7
Living situation, n	
Live with family	4
Live alone	4
Hemiplegic side, n	
Left	7
Right	1
Both	1
Time since stroke (months) mean (SD)	50.9 (21.5)

**Table 2.** Collective adaptive Intrinsic Motivation Inventory results with averages.

Question	P1	P2	P3	P4	P5	P6	P7	Mean (SD)
I enjoyed using this system very much.	7	7	7	6	7	6	6	6.57 (0.53)
I think I am pretty good at using this system.	6	7	7	1	6	5	6	5.43 (2.07)
I put a lot of effort into using this system.	6	6	7	3	7	6	6	5.86 (1.35)
I did not feel nervous at all whilst using it.	7	7	7	7	4	4	4	5.71 (1.60)
I thought using this system was a boring. (R)^a^	7	7	7	7	7	6	7	6.86 (0.38)
I believe this system could be of some value to me.	6	7	7	2	7	6	7	6 (1.83)
I believe I had some choice about using this system.	7	7	7	3	6	6	6	6 (1.41)
I didn’t try very hard to do well at using the system. (R)	7	7	7	7	6	5	2	5.86 (1.86)
I think that using this system is useful for tracking my rehabilitation exercises.	7	7	7	7	6	6	7	6.71 (0.49)
This system did not hold my attention at all. (R)	7	7	7	7	7	6	7	6.86 (0.38)
I felt very tense whilst using the system.	1	1	1	1	4	4	1	1.86 (1.46)
I felt like it was not my own choice to do this task. (R)	7	7	7	7	4	4	4	5.71 (1.60)
I thought this system was quite enjoyable	6	7	7	7	6	6	7	6.57 (0.53)
I would be willing to use this again because it has some value to me.	7	7	7	7	6	6	7	6.71 (0.49)
I am satisfied with my performance at using the system.	6	7	7	7	5	5	6	6.14 (0.90)
It was important to me to do well at using the system.	6	6	7	6	5	5	6	5.86 (0.69)
I used this system because I wanted to.	6	7	7	6	6	6	7	6.43 (0.53)
Using the system was an activity that I couldn’t do very well. (R)	7	7	7	7	7	6	7	6.86 (0.38)
I felt pressured while doing this.	1	1	1	1	1	2	1	1.14 (0.38)
I think doing this activity could help me to motivate myself in continuing my rehabilitation.	7	7	7	7	7	6	7	6.86 (0.38)

a R: reverse scored.

**Table 3.** System Usability Scale questions with average results; each item was rated on a 5-point Likert scale.

Question	Results, mean (SD)	
I think I would like to use this system frequently in the cohort.	4 (0)	
I found the system unnecessarily complex.	3.86 (0.35)	
I thought the system was easy to use.	3.71 (0.45)	
I think that I would need the support of a technical person to be able to use this system.	2.71 (1.75)	
I found the various functions in this system were well integrated.	3.57 (0.49)	
I thought there was too much inconsistency in this system.	3.57 (0.49)	
I would imagine that most people would learn to use this system very quickly.	3.86 (0.35)	
I found the tool very cumbersome to use.	3.57 (0.73)	
I felt very confident using the system.	3.86 (0.35)	
I needed to learn a lot of things before I could get going with this system.	3.86 (0.35)	

### Phase 2: Feasibility and Validity

Throughout the 6-month duration, 15 participants were recruited and completed the study (Table 4). Across the 15 participants, 207 activities were recorded by both digital and manual methods with an average number of sessions during inpatient stay of 9.1 (SD 7.4). There was a generally high agreement between the methods (*t*_206_=−1.6; *P*=.11; Cohen *d*=−0.06), confirming the equivalence of the 2 tracking methods. The overall analysis, encompassing all recorded data from the various rehabilitation stations and gym attendance, revealed an average absolute mean time difference of 1.23 (SD 11.01) minutes across 207 total uses. This absolute mean time difference reflects a small 1‐2-minute discrepancy between the digital and manual recorded times, indicating that whilst minor differences existed, they were not systematic and likely stemmed from normal variations in manual documentation.

**Table 4.** Participant characteristics of feasibility and validity trial.

Characteristics	All participants (n=15)
Age (years), mean (SD)	70.93 (13.31)
Sex, n	
Male	7
Female	8
Stroke type, n	
Ischemic	13
Hemorrhagic	2
Time since stroke (days), mean (SD)	19.5 (14.99)

**Figure 4.**
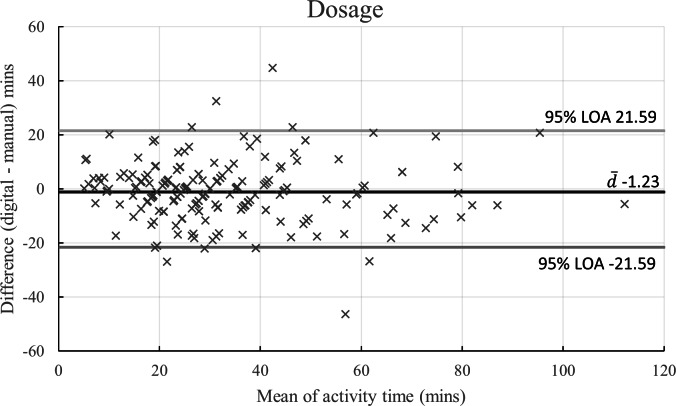
Bland-Altman plot comparing digital and manual dosage tracking methods. This plot shows the difference between the digital and manual dosage tracking methods for all recorded activities (n=207) at the stroke ward, plotted against the mean activity time. The plot includes the mean difference (bias, $\overset{-}{d}$) and the upper and lower limits of agreement at a 95% CI. The bias value, closely aligned with the x=0 axis, indicates a high degree of validity between the 2 methods. LOA: limit of agreement.

The Bland-Altman plot (Figure 4) shows an even spread across the range of activity durations, indicating consistent agreement between the digital tracker and manual recordings. The plot reveals a bias of −1.23 minutes, suggesting good accuracy of the digital tracker relative to the manual method, with only a small systematic bias. The LOA were calculated to be from −21.59 to 21.59 minutes, which encompasses most of the observed discrepancies. Despite this range, the review of activity-specific durations revealed only minor differences, no greater than 6 minutes on average (Table 5), further supporting the reliability of the digital tracking system.

**Table 5.** Summary of rehabilitation workstations and mean time discrepancies between digital and manual recordings. This table details the number of uses, the equipment involved, and the average time discrepancy (SD) in minutes between the digital tracker and manual recordings for each workstation. The “overall” row shows the combined data across all workstations and gym attendance.

Workstation category	Equipment	Number of uses, n	Time discrepancy (minutes), mean (SD)
Upper limb station	Graded Repetitive Arm Supplementary Programme (GRASP) kits, Gripable	24	5.23 12.8)
Cognitive applications	TinyTablet	24	2.73 (6.52)
Speech applications	TinyTablet	3	5.26 (21.08)
Power assist equipment	Three MOTOmed devices	67	3.23 (9.87)
All equipment uses	All above-listed equipment (excluding gym attendance)	118	0.27 (10.72)
Total time spent in rehabilitation gym	Attendance in gym	89	4.5 (14.55)
Overall	—^a^	207	1.23 (11.01)

a Not applicable.

## Discussion

### Principal Findings

The aim of this study was to evaluate a novel digital dose tracker against the standard manual recording method. Primary results showed a high level of agreement between the digital and manual methods, with Cohen *d* indicating a negligible effect size, which suggests that the differences between the 2 methods are minimal. This result confirms the digital tracker’s consistent and complete data capture. Manual recording is currently the method used for auditing and developing stroke care pathways [8]. The digital tracker’s ability to collect a broader range of activity data, as shown in Figure 4, highlights its potential to describe the intervention in greater detail, benefiting health care providers attempting to improve their services and researchers aiming to better understand response to rehabilitation interventions.

Phase 1 outcomes demonstrated the device’s clear usability among users. The high scores for SUS and IMI were not surprising given the preceding co-creation process. Usability is a critical factor in health care and rehabilitation settings, as it directly influences the likelihood of a device being adopted in routine practice. The high usability demonstrated by this digital dose tracker ensures that both health care providers and people with stroke history can integrate the device into daily operations without significant disruptions or extensive training. This ease of use supports seamless implementation in clinical environments, enhancing the overall user experience and increasing the likelihood of widespread adoption [22]. In rehabilitation, where consistent and accurate use of technology can significantly impact patient outcomes and care efficiency, high usability is particularly important [23].

Whilst the mean time discrepancy between the digital and manual methods is relatively small (1.23 min), the larger SD 11.01 min indicates some variability in individual time recordings. This variability could be attributed to factors such as differences in manual recording practices among therapists, variations in task complexity, and individual participant behavior during rehabilitation sessions. Despite this variability, the overall agreement between the 2 methods remains strong, suggesting that the digital system is a valid tool for tracking rehabilitation dosage, especially when considering the average results across multiple sessions and participants.

The digital tracker accurately tracked rehabilitation activities by logging the unique identifiers of both participants and devices, ensuring precise data collection. Conducted under direct observation, therapists manually recorded the time spent at each station, reflecting standard clinical practice. Whilst a minor average discrepancy of about 1 minute was noted between the digital system and manual recordings, this difference was minimal and fell within an acceptable range.

In line with the definition of rehabilitation dosage, which includes the intensity, frequency, and duration of rehabilitation activities, the digital tracker successfully captures the essential elements of dosage as required by this definition. Although intensity refers to the individualized adjustment of daily therapy across various domains such as physiotherapy, occupational therapy, and speech therapy, this tracker is particularly adept at accurately recording the frequency and duration of therapy sessions. By logging the start and stop times of each session, the tracker provides a measure of the total duration of rehab, which is critical for ensuring that people with stroke history receive the appropriate amount of therapy prescribed to them and can be used to monitor achievement of guidelines.

The digital trackers approach to tracking rehabilitation dosage mirrors the standard clinical practice where therapists estimate the total time a patient spends at a therapy station. Therapists typically log this time without distinguishing between active therapy and brief pauses, which has been an accepted method in clinical practice for years. The 1-minute average discrepancy observed between the therapists’ manual logs and the digital recordings reflects the minor variations inherent in manual estimation. Importantly, the fact that these times align so closely and fall within clinically acceptable margins of error demonstrates that this method does not negatively affect the results. This small difference is within the clinically acceptable range, reinforcing the digital trackers reliability as a tool for tracking rehabilitation dosage.

The distribution of attendance across the different rehabilitation workstations also provides insight into the priorities and preferences of people with stroke history during their rehabilitation. Users at the stroke ward’s rehabilitation gym had the autonomy to select the rehabilitation they preferred, with guidance and supervision, during their sessions. The Power Assisted Equipment station had the highest number of uses (n=67) which may reflect a common patient priority of focusing on motor recovery [24]. This preference is often driven by the desire to regain physical independence, which has been previously recognized as a priority for individuals with stroke over cognitive or speech rehabilitation.

The Bland-Altman plot, Figure 4, showed that the device was accurate in depicting a user’s rehabilitation journey and the LOA were calculated with a 95% CI. There is currently no clinical documentation which specifies an acceptable LOA, but a margin of error within ±5%‐10% was deemed acceptable, given that manual recording in rehabilitation contexts could vary widely, affecting the reliability of data [25]. The LOA determined in this study was within this range.

Table 3 shows that 3 rehabilitation workstations had an average time difference of just under 6 minutes. This discrepancy is attributed to the need for hoist or transfer assistance for users with low to no mobility, resulting in waiting periods. This also applies to the time taken for participants to transfer in and out of the rehabilitation gym. Overall, wide differences were rare, and outliers were attributed to instances of human error in the manual recordings when the therapist was observing and recording multiple participants at once.

The feasibility of the digital dose tracker was further demonstrated by the fact that all users of the rehabilitation gym, regardless of their varying levels of cognitive or physical impairment, were able to effectively use the system. There were no withdrawals in Phase 2 or errors recorded by the tracker during the study, nor were any observed by the 2 clinical staff, demonstrating the trackers accessibility and functionality across a diverse stroke population.

Whilst the dosage tracker can operate on its own, it is a subcomponent of a more complex device with the intended purpose to measure both dosage and intensity of a person’s rehabilitation. This device is a prototype and proof of concept, throughout the phase 1 usability trial participants were allowed to give feedback about the device to allow for adjustment and modifications, this included adjusting the brightness of the LEDs and increasing the volume of the speaker to improve audio and visual feedback. The device is limited in the quality of its components due to funding however if additional funding were to be obtained this would contribute to the refinement of the trackers to reduce the size and thus improve portability.

The study faced challenges related to the rate of participant uptake. A significant number of potential participants could not be included because they were only in the hospital for a few days, which did not allow sufficient time for the consent process. In addition, the rate of uptake in the study and the number of viable participants varied depending on the number of people admitted to the stroke ward during the study period. This limitation reduced the overall sample size, impacting the generalizability of the findings. Future studies could consider running for a longer duration to accommodate the consent process and variations inpatient admissions, thereby ensuring a more comprehensive assessment of rehabilitation dosage.

### Conclusion

This study has evaluated a novel, co-designed, digital system for monitoring rehabilitation dose finding it largely agreed with traditional manual methods. It is one of the first studies to implement such a method of rehabilitation dosage tracking in a clinical rehabilitation setting. This promising system has the capacity to provide an accurate and automated method for measuring achievement of the new National Clinical Guidelines for Stroke recommendations and providing practical support for therapists and researchers.

## Abbreviations

IMI: Intrinsic Motivation Inventory
IoT: Internet of Things
LOA: limits of agreement
NFC: Near-Field Communication
NHS: National Health Service
NICE: National Institute for Health and Care Excellence
SSNAP: Sentinel Stroke National Audit Programme
SUS: System Usability Scale
